# Hepatitis C virus genotypes circulating in district Swat of Khyber Pakhtoonkhaw, Pakistan

**DOI:** 10.1186/1743-422X-8-16

**Published:** 2011-01-14

**Authors:** Inamullah, Muhammad Idrees, Habib Ahmed, Sajid-ul-Ghafoor, Muhammad Ali, Liaqat Ali, Aziz Ahmed

**Affiliations:** 1 Department of Genetics, Hazara University Mansehra, Pakistan; 2 National Centre of Excellence in Molecular Biology, University of the Punjab, Lahore, Pakistan; 3 Department of Medicine, Saidu Teaching Hospital, Saidu Sharif, Swat, Pakistan; 4 Division of Molecular Virology, National Centre of Excellence in Molecular Biology, University of the Punjab, Lahore, Pakistan

## Abstract

Hepatitis C virus (HCV) is the leading cause of chronic hepatitis worldwide and its subtypes/genotypes are clinically important for clinical management and vaccine development. The present study describes frequency distribution of different HCV genotypes and their treatment status in HCV RNA positive patients from district Swat. A total of 185 HCV infected sera were analyzed by molecular genotyping assay. The most prevalent genotype was 3a (34.1%), followed by 2a (8.1%), 3b (7%) and 1a (5.4%). The samples found untypable by the present method of genotypes was 37.8% while, patients with mixed genotype infections were 7.6%. More than 80% of untypable cases were from those HCV patients who had received interferon plus ribavirin standard therapy in the past and either were non-responders and were relapsed thereafter or were under treatment. In conclusion, genotype 3a is the most prevalent HCV genotype in the region. A high prevalence rate of untypable genotypes is present in treated patients that need further investigation for the successful genotyping by developing new assays or using viral sequencing method.

## Background

Hepatitis C virus (HCV) is a positive single strand RNA virus of Flaviviridae family [1,2] that is approximately 9.6 Kb in length having a 5' non-coding region (5'NCR), a long open reading frame (ORF) encoding a polyprotein precursor of 3,000 amino acids and 3'NCR. Functions of 5'NCR as an internal ribosomal entry site (IRES) is important for cap-independent translation of the viral RNA [3]. The virus infect hepatocytes and B lymphocytes of human cells [4,5], and produce 10 trillion virus particles per day, even in the late phase of infection [1]. Viral infection is the major cause of liver cirrhosis in approximately 20% of patients that after 10 years lead to hepatocellular carcinoma (HCC) in a subset of incidence of 3%per year [6]. The infection is also associated with cryoglobulinaemia [7] non-Hodgkin's lymphoma [8] and glomerulonephritis [9]. There are 200 million people of the world population are infected with HCV [10] of which the seroprevalence rate is about 1% in North America, Mediterranean and in Asian countries it is about 3-4%, while in central Africa and Egypt it is 10-20%[11,12].

Globally six major Hepatitis C virus (HCV) genotypes and multiple subtypes have been identified and are generally studied for epidemiology, vaccine development and clinical management of the infection [13]. Interferon in combination with ribavirin is the only available approved treatment for chronic hepatitis C [14]. In Western patients the initial responses have been reported to be between 40-70% but sustained response was noted in only 10-25% after treatment [15]. Evidence shows that HCV genotype 2 and 3 infected patients are more likely to have a sustained virological response to anti-viral treatment than genotype-1 infected patients [16]. Combine treatment with interferon and ribavirin have 65% and 30% sustained virological response to HCV genotype 2, 3 and 1 respectively [17,18].

The worldwide distribution and relative prevalence of these three HCV genotypes varies from one geographic region to another. In the United State of America and Europe the most common subtypes are 1a and 1b [19-21], While subtype 1b is most commonly found in Japan [22]. HCV Subtype 2a and 2b are mostly found in North America, Europe, and Japan while 2c is found only in northern Italy [16,20-22]. In North Africa and the Middle East the most common genotype is 4 while genotype 5 and 6 are found in South Africa and Hong Kong, respectively [23,24]. Genotypes 7, 8, and 9 have been reported only in Vietnamese [25] and genotypes 10 and 11 are commonly found in Indonesian patients [26]. It has been proposed that genotypes 7 through 11 should be consider as variants of the same group and classified as a single genotype, type 6 [27,28].

On the distribution of hepatitis C virus genotypes, only a few numbers of studies are available from Punjab and Sindh provinces of Pakistan [29-31]. The frequency distribution of various HCV genotypes in Khyber Pakhtoonkhaw (KPK) are recently reported in one study [13] in which HCV genotypes 1a, 1b, 3a and 3b are commonly found in various parts of KPK among these genotypes 3a is frequently available [13]. Study on the molecular genotyping of HCV prevalent in KPK is limited. Therefore, this study was conducted to find out the molecular epidemiology of various HCV genotypes and subtypes present in Swat district of KPK.

## Methods

### Subjects

A total of 185 patients infected with Hepatitis C virus were selected for HCV Genotyping. Informed consent was taken from each infected patient, containing age, sex, demographic characteristic, apparent mode of disease transmission, area/district, and estimated time of infection along with complete address and contact numbers of the patients. Blood samples were collected from patients by visiting hospitals in different area of swat.

### Qualitative PCRs for HCV RNA

Reverse transcriptase (RT) PCR described by idress et al [30] was done for the detection of HCV RNA. RNA was isolated from 100 μl patient's sera using Quigen RNA extraction kit according to the kit protocol. Nested PCR were performed using Taq DNA polymerase enzyme (Fermentas Technologies USA) in a volume of 20 μl reaction mix. The nested PCR products were visualized on 2% agarose gel.

### Genotyping of HCV

Type-specific HCV genotyping procedure as described previously in detail [31] was used. About 10 μl of HCV RNA was reverse transcribed to cDNA using 100 U of M-MLV RTEs at temperature of 37°C for 50 minutes. Two μl synthesized cDNA was used for PCR amplification of 470-bp region from HCV 5'NCR along with core region by first round PCR amplification. The amplified first round PCR product were subjected to two second rounds nested PCR amplifications. One with mix-I primers set and the second with mix-II primers set in a reaction volume of 10 μl mix-I had specific genotype primers set for 1a, 1b, 1c, 3a, 3c and 4 genotypes and mix-II contain specific genotype primers set for 2a, 2c, 3b, 5a, and 6a genotypes.

After second round of PCR, the products were separated by 2% agarose gel and ethidium bromide was used for staining to visualize the gel under UV transilluminator. A DNA ladder of 100-bp (Invitrogen, Corp., California, USA) was run in each gel as DNA size marker and the HCV genotype for each sample was confirmed by HCV genotype-specific PCR band. Gel documentation system (Geldoc System, Eppendorf Inc, Germany) was used for gel photograph. The sensitivity of the assay is about 250 IU per ml sample and specificity is 97%.

### Statistical analysis

Data was analyzed and summarize by using statistic software SPSS version 16.0 for windows. ANOVA and t-test was applied to assess the statistical significance of the data. The data was obtainable as mean values or number of patients. P-value less than 0.05 was considered as significant.

## Results

Total of 185 HCV positive samples from district Swat were selected for HCV genotype analysis. Among them 105 (56.8%) were males and 80 (43.2%) were females with 171 (92.4%) married and only 14 (7.6%) unmarried patients. Mean age of the patients was 34.72 years. Most of the patients had no previous history of drug addiction, only 17 (9.2%) and 25 (13.5%) of the patients were smokers and snuff users respectively while, no patient was observed with injection drug use.

Table 1 shows age wise distribution of different genotypes prevalent in District Swat. Total no of patients successfully genotyped were 115 (62.16%). Total number of HCV 3a genotype samples were 63 (34.1%), 2a (8.1%), 3b (7%) and 1a (5.4%). Mixed infections of two different genotypes were observed in 14 (7.6%) while 70 (37.8%) of the patients were untypable by the mentioned method. This shows that most prevalent genotype is 3a followed by 2a, 3b and 1a. High HCV infection rate of 42.7% was observed in the age group of 20 to 30 years while only 0.5% of the patients were of the age above 60 years. The HCV infection was found more prevalent in married patients (92.4%) as compared to non-married persons.

**Table 1:** Age wise distribution of the patients genotyped

Age in years	Genotype						
	
	3a	2a	1a	3b	Untypable	Mixed	Total
10-20	1	1	0	0	4	0	6 (3.24%)

20-30	27	5	5	8	26	8	79 (42.7%)

30-40	18	3	2	2	20	3	45 (24.32%)

40-50	13	4	2	3	17	2	41 (22.16%)

50-60	3	2	0	0	1	1	7 (3.78%)

>60	1	0	1	0	2	0	4 (2.16%)

Total	63	15	10	13	70	14	185

Table 2**shows **successfully genotyped and non-genotyped samples based on anti-viral treatment status of the patients. Of the total 70 untypable cases, 58 (47.9%) were from those patients who had received or were receiving interferon plus ribavirin standard therapy in the past and either were non-responders or were relapsed thereafter and only 1 (9.1%) was from untreated group. Those patients who were unaware about their treatment status in the past, 11 out of 53 (20.8%) were found with untypable genotypes.

**Table 2:** Successfully genotyped and non-genotyped samples based on anti-viral treatment status

Treatment Status	Number of patients samples	Successfully genotyped (%)	Found untypable (%)
Previously received interferon plus ribavirin therapy	121	63 (52.1)	58 (47.9)

Never received interferon plus ribavirin therapy	11	10 (90.9)	1 (9.1)

Unknown about treatment	53	42 (79.2)	11 (20.8)

Different mode of contamination of various HCV genotypes is clear from table 3. In 26.48% of the HCV infected population the mode of contamination was surgeries, in 12.4% was blood transfusion, 10.8% (pregnant females) got infection during deliveries and 10.3% had visited foreign countries especially Afghanistan in past and probably got infected over there. The mode of contamination was unknown in 40.0% of the infected individuals.

**Table 3:** Status of the patient Infectious Detection * Genotype Cross tabulation

Infectious Detection	Genotype						Total
		
	3a	Untypable	2a	Mixed	1a	3b	
HCV positive (Surgical Patients)	15	20	6	3	2	3	49 (26.48%)

HCV positive (Pregnant women)	7	10	2	0	1	0	20 (10.8%)

HCV positive (Blood Donors)	9	7	1	3	3	0	23 (12.4%)

Unknown	26	26	5	5	3	9	74 (40.0%)

HCV positive (Foreign screeners)	6	7	1	3	1	1	19 (10.3%)

Total	63	70	15	14	10	13	185

Results of the multivariate ANOVA showed that the genotype prevalence is independent of age of the patient (p = 0.879), gender (p = 0.214) and marital status (p = 0.291) at 95% confidence interval (α = 0.05). Similarly, treatment status of the patients were also shown to be independent of age, gender and marital status (p > 0.05) at 95% CI. Analysis of paired sample t-test showed that all the six different genotypes are independent of age of the patients (p > 0.05) at CI of 95%.

## Discussion

Swat is a district with a population of 12'57'602 (according to 1998 censuses) located in the Khyber Pakhtoonkhaw (KPK) province of Pakistan [32]. Hepatitis C virus seropositivity in Swati population was reported to be 13.8% while HCV RNA positivity was 4.7% [33]. In this study, 185 HCV RNA positive samples were analyzed for genotyping. The most prevalent genotype was 3a (34.1%), followed by 2a (8.1%), 3b (7%), 1a (5.4%) while, mixed genotype infection were 7.6%. The rate of untypable genotype was 37.8% in the current study. Recently Ahmad et al. [33] investigated the prevalence of different genotypes in district Swat and found 49.5% prevalence of 3a genotype, 3b (3.2%), mixed genotypes infection (8.7%) and untypable (3.6%) [13]. Ali et al. [33] genotyped 415 HCV positive patients from KPK province and reported 3a (57.83%), 3b (6.2%), 1a (072%), mixed genotypes (6.73%) and untypable (27.95%). The authors of both the studies described above were unable to establish the status of treatment in their enrolled patients. In another study from Lahore (a populated city in Pakistan), Ahmad et al. [34] reported 3a (55.9%), 3b (3.2%), 1a (23.6%), mixed genotypes (1.2%) and untypable (2.5%). A more detailed study from Pakistan showed the prevalence of 3a (62%), 3b (9%), 1a (3%), 2a (2.144%), mixed (4.718%) and untypable (17.16%) [34]. The present study shows high prevalence of 2a of 8.1% genotype in Swati population as compared to that reported from the rest of the country. Genotypes 2a is common in Europe, North America and Japan [35] which suggest that this genotype may be introduced due to frequent traveling of the local population to these countries.

The high percentage of untypable HCV genotype seen in the current study is due to the status of patients samples used for HCV genotyping as 82.9% of our untypable cases were from those HCV patients who had received interferon plus ribavirin standard therapy in the past and either they were non-responders or initially responded to therapy (end of treatment response) but later on were relapsed. It is convincing that this high number of samples with untypable genotypes is due to mutations and emergence of new quasispecies that emerged during therapy due to drugs pressure. HCV mutates very rapidly during its replication cycle that permits for the emergence of drug resistance mutations that is very common. Rapid emergence of new resistance strains/quasi-species occurs in a population of viruses already present, allowing the new/resistant virus to become the leading strain, therefore, in order to correctly genotype these newly emerged strains, clinical laboratories may require to up date their genotyping assays as the previously developed assays may not be able to designate a specific genotype/subtype to these strains.

## Conclusion

We conclude that (i) HCV genotypes 3a is the most prevalent genotype in district Swat (ii) The current genotyping methods fail to genotype the newly emerged strains (iii) Major mode of HCV transmission is unknown.

## Abbreviations

HCV: hepatitis C virus; M-MLV: Molony-murine leukemia virus; KPK: Khyber Pakhtoonkhaw; ABI: Applied Biosystem Inc.; RT-PCR: reverse transcriptase polymerase chain reaction; cDNA: complimentary DNA.

## Competing interests

The authors declare that they have no competing interests.

## Authors' contributions

AA & HA conceived the study, participated in its design and coordination and gave a critical view of manuscript writing. IU collected epidemiological data, performed genotype analysis and analyzed the data statistically. MI helped IU in molecular genotyping assays and gave a critical view of manuscript writing and participated in data analysis. SUG, MA, LA and AA helped IU in data analysis. All the authors read and approved the final manuscript.
